# Tuberculosis as an Army Problem

**Published:** 1919-01

**Authors:** 


***Tuberculosis as an Army Problem.*** By Surgeon LAWRASON BROWN and Major JOSEPH H. PRATT. *The Military Surgeon,* August, 1918.

The authors report a brief review of the tuberculosis examination of the 76th division, together with criticisms of methods and suggestions for future work.

The examination board at Camp Devens consisted of 19 physicians, and examined 27,300 men. The whole board was under the direction of a president, who was responsible directly to the division surgeon. The rapid routine examinations were carried out by five groups of three members each, called " local boards ".

The examiners were not required to take clinical histories, but information was obtained in each case in the following way : mimeographic questionnaires, containing eight questions which, if truthfully answered, would bring to the knowledge of the board all symptoms of tuberculosis if they existed, or a past history, or a family history of that disease, were filled out by each man.

Among the 27,304 officers and men examined, 184 cases of pulmonary tuberculosis were brought to light (0.67 per cent.); 135 of these were discharged from the army and 48 retained in the service.

When a soldier was suspected of having pulmonary tuberculosis he was referred to the tuberculosis ward, and there carefully reexamined. When definite physical signs were found, and the radiograph was confirmatory, the man was rejected if the physical signs were those of a potentially active lesion, or of some extent i.e., to the third rib and fourth vertebral spine. If the lesion were less extensive and without râles, and the radiograph showed a parenchymateous lesion of considerable extent, the man was often rejected.

*Râles in Army Examination.* The way of qualifying the different varieties of râles was hopelessly confused at the beginning of the board's work. Each man qualified the râles as it seemed best to him. But soon the following division was quite generally adopted : (1) pleural friction; (2) rhonchi-sonorous and sibilant râles; (3) crepitant râles; (4) fine râles; (5) moderately coarse; (6) coarse; (7) cavernous.

The old law that apical conditions are usually tuberculous, and abasl, seemed to hold in the board's examinations. When the râles were apical, two-thirds of the men were rejected; when in the middle of the lung or at the base, only one-half were rejected. The importance of râles is shown by the fact that three times as many men with râles were rejected as those without râles.

Among a series of 150 cases of pulmonary tuberculosis, the sputum was found to be positive in only 10. The temperature was rarely elevated. It appears that most of the cases were inactive or healed. A great number of cases, who had never had symptoms, were discovered with signs of widespread involvement of the lungs.

During the early weeks of training, a large number of cases with active disease had been admitted to the base hospital, previous to the board's examination.

The authors suggest that fuller description of the examinations required should be furnished the examiners, and should include instructions somewhat as follows :

(1) Use ausculation only in first rapid examination.

(2) Listen to the breathing in four or five places at least, on each side, front and back, before using the auscultatory cough.

(3) Use the auscultatory cough whether the breathing is normal or abnormal.

(4) Any man with râles detected by two members of the board should be referred to the tuberculosis specialist for study.

(5) Attention should be called to the fact that too hard coughing obscures rather than helps the detection of râles.

Examiners should be able to check up their physical examinations with the X-ray.

More sputum examinations should be made.

Colonel Bushnell's terminology for the differentiation of the râles is highly recommended and should be adopted by every one.

